# ERCC1和GST-pi在肺癌中的表达及预后意义

**DOI:** 10.3779/j.issn.1009-3419.2010.03.02

**Published:** 2010-03-20

**Authors:** 崇安 许, 丹 冯, 琳 李, 萍 于, 雪君 胡, 云鹏 刘

**Affiliations:** 110032 沈阳，中国医科大学附属第四医院肿瘤内科; 110001 沈阳，中国医科大学附属第一医院肿瘤内科; 110001 沈阳，中国医科大学附属第一医院呼吸内科 Department of Oncology Medicine, the Fourth Hospital of China Medical University, Shenyang 110032, China

**Keywords:** 肺肿瘤, ERCC1蛋白, GST-pi蛋白, 免疫组织化学, Lung neoplasms, ERCC1 protein, GST-pi protein, Immunohistochemistry

## Abstract

**背景与目的:**

切除修复交叉互补基因1（excision repair cross complementing 1, ERCC1）和谷胱甘肽S-转移酶-Pi（glutathione S-transferase Pi, GST-pi）与肿瘤的发生与预后密切相关。本研究探讨ERCC1和GST-pi在肺癌中的表达与临床病理特征和预后的意义。

**方法:**

选取148例肺癌标本，与7例正常肺组织标本一起制成组织芯片，应用免疫组化S-P法检测ERCC1和GST-pi的表达，并与临床病理特征及预后进行比较分析。

**结果:**

ERCC1和GST-pi在肺癌标本中的阳性表达率分别为36.2%和73.6%，ERCC1和GST-pi在正常肺组织标本中均无表达，ERCC1阳性表达在非小细胞肺癌（non-small cell lung cancer, NSCLC）、分化程度为中高分化和吸烟指数 < 400的患者中明显升高（*P*值均 < 0.05），GST-pi阳性表达在无吸烟者及非小细胞肺癌患者中明显升高（*P*值均 < 0.05）。ERCC1和GST-pi的表达呈正相关（*r*=0.253, *P*=0.001）。*Kaplan-Meier*生存分析显示，ERCC1阳性表达者5年总生存率优于阴性表达者，ERCC1的表达与生存显著相关（*P*=0.037），GST-pi的表达与生存无显著相关（*P*=0.614）。*Cox*多因素分析结果显示，NSCLC患者中肿瘤大小（*P*=0.028, 95%CI: 1.087-4.378, RR=2.181）和临床分期（*P*=0.019, 95%CI: 1.076-2.279, RR=1.566）是影响预后的独立危险因素；ERCC1和GST-pi的表达不是影响预后的独立因素。

**结论:**

ERCC1和GST-pi在非小细胞肺癌中表达升高并且存在正相关关系，可能在非小细胞肺癌发生发展中起协同作用。ERCC1阳性表达者生存期长，可能在预后判定中有一定作用。

## Abstract (en)

**Background and objective:**

It has been known that the expression levels of ERCC1 and GST-pi were correlated with tumorigenesis and prognosis. The aim of this study is to investigate the relationship between expression levels of ERCC1 and GST-pi, and clinicopathologic parameters and survival in patients with lung cancer.

**Methods:**

The expression levels of ERCC1 and GST-pi were detected by immunohistochemical staining on tissue micro-array sections made of 148 cases of lung cancer and 7 cases of normal lung samples. The results were compared with relevant clinical and pathologic data.

**Results:**

Positive rates of ERCC1 and GST-pi were 36.2% and 73.6%, respectively. None of normal lung samples was positive staining. Positive expression of ERCC1 was significantly higher in group of non-small cell lung cancer (NSCLC), highly differ- entiated and the smokers less than 400 (*P* < 0.05), positive expression of GST-pi was significantly higher in group of non-smok- ers and NSCLC (*P* < 0.05). There were significant correlations between expression of ERCC1 and GST-pi (*r*=0.253, *P*=0.001). The 5 years survival rate was higher in positive expression of ERCC1. There was significant correlations between expression of ERCC1 and survival (*P*=0.037). There was no significant correlations between expression of GST-pi and survival (*P*=0.614). Multivariate analysis using *Cox* regression model showed that expression levels of ERCC1 and GST-pi were not the important independent prognostic factors for survival.

**Conclusion:**

ERCC1 and GST-pi are aberrant highly expressed in NSCLC with positive correlation, which indicate they might act synergistically in tumorigenesis of NSCLC. The positive expression of ERCC1 have better survival and may have effect on prognosis.

肺癌的发病率及死亡率不仅在欧美国家位居首位^[1]^，在我国也已成为城市居民恶性肿瘤死亡率的第一位^[2]^，其中非小细胞肺癌（non-small cell lung cancer, NSCLC）约占全部肺癌的80%^[3]^，由于肺癌起病隐匿，目前仍缺乏有效的筛查和早期诊断方法，患者出现症状时多为晚期，预后较差，总的5年生存率不超过15%，有症状者≤10%^[4]^。很多病人在接受根治性手术后仍会复发和转移，单凭临床病理分期已不能对预后做出准确评估，因此，有必要研究不同的分子标志物在肺癌发生发展及预后中的作用。

DNA损伤修复系统在维持基因组功能完整性、修复致癌因素所致的损伤以及抗癌过程中起到极为重要的作用^[5]^，切除修复交叉互补基因1（excision repair cross complementing 1, ERCC1）是DNA修复系统中的一个关键基因，在多种DNA损伤中发挥修复作用，与恶性肿瘤的发生、预后及治疗反应等密切相关。谷胱甘肽S-转移酶-Pi（glutathione S-transferase Pi, GST-pi）是谷胱甘肽S-转移酶家族成员之一，是体内生物转化最重要的Ⅱ相代谢酶，是细胞抗损伤抗癌变的重要解毒系统^[6]^。为了进一步研究与肺癌相关的分子标志物特点，我们应用组织芯片和免疫组化方法，检测148例肺癌标本中ERCC1和GST-pi的表达情况，并分析其表达与临床病理特征和预后的关系。

## 1 材料和方法

### 1.1 材料

收集中国医科大学附属第一医院和中国医科大学附属第四医院1997年1月-2004年12月外科手术切除并有完整临床资料的肺癌石蜡包埋标本148例，男性104例，女性44例，年龄34岁-76岁，中位年龄58.52岁，有吸烟史93例，无吸烟史55例；病理类型为小细胞肺癌（small cell lung cancer, SCLC）22例，NSCLC 126例，NSCLC中鳞癌50例，腺癌61例，腺鳞癌1例，类癌1例，大细胞癌6例，多形性癌7例；分化程度为高分化20例，中分化59例，低分化41例，未分化28例；根据UICC1997年新的修订标准进行TNM分期：Ⅰ期56例，Ⅱ期25例，Ⅲ期67例；淋巴结转移阳性者83例，淋巴结转移阴性者65例；所有患者术前均未接受过化疗、放疗或免疫治疗。对照组标本选取7例远离肿瘤组织（> 5 cm）的正常肺组织。

### 1.2 方法

#### 1.2.1 组织芯片制作

将NSCLC和正常对照标本制作成组织芯片（技术由上海芯超生物科技有限公司提供），简要制作步骤如下：存档切片经病理专家复诊，并进行组织学定位后，再选取对应的蜡块，以备制作组织芯片使用。应用组织阵列仪打孔取材（直径1.0 mm），每块取两点，制作成阵列块；应用切片机连续切片，厚度为4 μm；捞片，烤片及染色后再次复诊，制作成可供免疫组化染色使用的组织芯片数张。

#### 1.2.2 免疫组织化学染色

采用免疫组化S-P方法检测ERCC1和GST-pi的表达，实验步骤按试剂盒说明书进行。ERCC1鼠抗人单克隆抗体浓缩型购自美国Neomarkers公司，工作浓度为1:100，GST-pi鼠抗人多克隆抗体即用型及SP免疫组化试剂盒购自福建迈新生物技术开发有限公司，选用提供的阳性切片在同一条件下染色作为阳性对照，用PBS代替一抗作为阴性对照。

### 1.3 结果判定

染色结果由两名有相关经验的研究者应用HPIAS-1000高清晰彩色病理图文分析系统独立进行判定，随机选取5个高倍视野（×400）计数100个肿瘤细胞中阳性细胞数，意见不同时协商解决。ERCC1以胞核中出现明显的棕黄色颗粒为阳性细胞，阳性细胞数≥10%记作阳性（+），无阳性细胞或阳性细胞 < 10%记为阴性（-）。GST-pi以胞质和（或）胞核出现棕黄色颗粒为阳性细胞，阳性细胞数≥20%记作阳性（+），无阳性细胞或阳性细胞 < 20%记为阴性（-）。

### 1.4 统计学分析

采用SPSS 13.0软件进行数据分析，率的比较采用*χ*^2^检验，ERCC1和GST-pi之间表达的相关性采用*Spearman*秩和相关分析；生存数据分析采用*Kaplan- Meier*法，组间比较采用*Log-rank*检验；对影响生存预后的联合效应采用*Cox*比例风险模型进行多因素分析，纳入的变量为年龄、性别、吸烟、病理类型、分化程度、肿瘤大小、淋巴结转移、ERCC1和GST-pi的表达；*P* < 0.05为有统计学差异，所有检验和*P*值均为双侧。

## 2 结果

### 2.1 ERCC1和GST-pi在肺癌和正常肺组织中的表达

148例肺癌中（图 1A，图 1C），ERCC1阳性（图 1B，图 2A）56例（37.8%），GST-pi阳性（图 1D，图 2B）109例（73.6%），而7例正常肺组织中二者表达均为阴性。肺癌标本中ERCC1（*P*=0.042）和GST-pi（*P* < 0.001）的表达明显高于正常肺组织。

**1:**
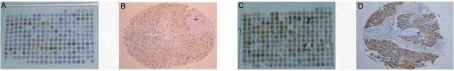
肺癌组织芯片及一个点的ERCC1和GST-pi的阳性表达 The tissue microarray of lung cancer and the positive expressions of ERCC1 and GST-pi in one site of lung cancer tissue

**2:**
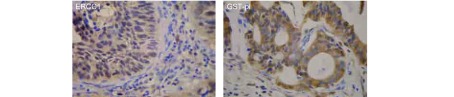
ERCC1和GST-pi在非小细胞肺癌组织中的表达（SP，×400） The expressions of ERCC1 and GST-pi in NSCLC tissue (SP, ×400)

### 2.2 ERCC1和GST-pi的表达与肺癌临床病理特征的关系

ERCC1阳性表达在NSCLC、中高分化和吸烟指数 < 400的患者中明显升高（*P*值分别为0.039、0.016和0.049），GST-pi阳性表达在无吸烟者（*P*=0.034）及NSCLC患者（*P* < 0.001）中明显升高，二者表达与患者年龄、性别、肿瘤大小、淋巴结转移及TNM分期均无关（*P*值均大于0.05）（表 1）

**1:** ERCC1和GST-pi的表达与肺癌患者临床病理特征的关系 The relationship between ERCC1 expression, GST-pi expression and clinical pathophysicological characteristics of lung cancer patients

Characteristics	ERCC1 expression						GST-pi expression				
	Cases	Positive	Positive rate (%)	*χ*^2^	*P*		Cases	Positive	Positive rate (%)	*χ*^2^	*P*
Age (year)				2.992	0.084					1.195	0.274
< 65	36	18	50.0				36	24	66.7		
>65	112	38	33.9				112	8S	75.9		
Sex				0.251	0.616					1.122	0.290
Male	104	38	36.5				104	74	71.2		
Female	44	18	40.9				44	35	79.5		
Smoking status				2.159	0.142					4.499	0.034
Non-smokers	55	25	45.5				55	46	83.6		
Smokers	93	31	33.3				93	63	67.7		
Cigarette smoking index				3.867	0.049					1.469	0.226
< 400	88	39	44.3				88	68	77.3		
>400	60	17	28.3				60	41	68.3		
Histology				4.245	0.039					23.299	<0.001
Small cell lung cancer	22	4	18.2				22	7	31.8		
Non-small cell lung cancer 26		52	41.3	0.703	0.402		126	102	81.0	0.505	0.477
Squamous carcinoma	50	19	38.0				50	42	84.0		
Adenocarcinoma	61	28	45.9				61	48	78.7		
Differentiation grade				5.832	0.016					3.247	0.072
Pooriy and undifferentiated 69		19	27.5				69	46	66.7		
Moderately and highly	79	37	46.8				79	63	79.7		
Size of the tumor				2.451	0.117					0.569	0.451
T1+T2	111	46	41.4				111	80	72.1		
T3+T4	37	10	27.0				37	29	78.4		
Lymphatic metastasis				0.297	0.586					0.180	0.671
Positive	65	23	35.4				65	49	7S.4		
Negative	83	33	39.8				83	60	72.3		
TNM stage				0.569	0.451					1.166	0.280
Ⅰ+Ⅱ	111	80	72.1				83	64	77.1		
Ⅲ	37	29	78.4				65	45	69.2		

### 2.3 ERCC1和GST-pi表达的相关性

*Spearman*秩和相关分析结果显示，ERCC1和GST-pi的表达呈正相关（*r*=0.214, *P*=0.009）。

### 2.4 ERCC1和GST-pi的表达与肺癌患者预后的关系

148例肺癌病人的5年总生存率为37.4%，*Kaplan-Meier*生存分析显示，ERCC1阳性表达者5年总生存率优于阴性患者（*P*=0.037）（图 3A），分层分析发现，NSCLC患者中ERCC1阳性患者的5年生存率明显高于ERCC1阴性患者（*χ*^2^=4.058, *P*=0.044）（图 3B），而SCLC患者中ERCC1的表达对生存无明显影响（*χ*^2^=0.225, *P*=0.635）。进一步分析显示，Ⅰ期和Ⅱ期患者的5年生存率显著优于Ⅲ期患者（*χ*^2^=17.427, *P* < 0.001）（图 3C），并且Ⅰ期和Ⅱ期患者ERCC1阳性的的5年生存率亦显著优于Ⅲ期ERCC1阳性患者（*χ*^2^=17.849, *P* < 0.001）（图 3D）。GST-pi表达阳性与否对5年生存率无明显影响（*P*=0.614），分层分析显示无论NSCLC还是SCLC患者GST-pi的表达对生存均无明显影响（*P*均 > 0.05）。分析ERCC1和GST-pi都表达或都不表达或只有一种表达之间5年生存率的差异，结果显示无统计学差异（*χ*^2^=1.303, *P*=0.254）

**3:**
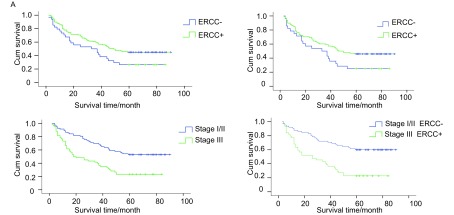
患者的生存曲线 The *Kaplan-Meier* survival curves of lung cancer patients

*Cox*多因素分析结果显示，NSCLC患者中肿瘤大小（*P*=0.028, 95%CI: 1.087-4.378, RR=2.181）和临床分期（*P*=0.019, 95%CI: 1.076-2.279, RR=1.566）是影响预后的独立危险因素；ERCC1和GST-pi的表达不是影响预后的独立危险因素。在SCLC患者中，肿瘤大小、临床分期、淋巴结转移及ERCC1和GST-pi的表达等均不是影响预后的独立危险因素。

## 3 讨论

*ERCC1*是DNA修复基因，定位于19号染色体上，是核苷酸剪切修复家族中的一个重要成员，编码297个氨基酸，与XPF形成异源二聚体，在DNA单链受损处的5’端进行剪切而发挥功能。研究证实DNA修复基因的修复能力与肺癌的发病和耐药有关，DNA修复基因修复力低者肺癌发生的风险较正常人明显增加，而修复能力高者常对化疗药物耐药，使化疗失败^[7-9]^。

我们^[10]^曾检测了ERCC1在116例NSCLC和12例癌旁肺组织中的表达，阳性率分别为40.5%和16.7%，差异无统计学意义，同时发现ERCC1的表达与肺癌分化程度、肿瘤大小和TNM分期密切相关，ERCC1阳性表达组5年生存率高于阴性表达组。Simon等^[8]^检测了51例NSCLC患者术后癌组织中的ERCCI表达，发现ERCC1的表达与吸烟情况无关。本文的研究发现ERCC1在肺癌中的表达高于正常肺组织（*P*=0.042），并且ERCC1表达在吸烟指数小于400的患者中明显升高（*P*=0.049），上述研究结果的差异分析与样本来源不同有关。目前有关ERCC1在肺鳞癌及腺癌中的表达情况研究尚有争议，Simon等^[8]^的研究结果显示ERCC1在腺癌中的表达高于鳞癌，而Wachters等^[11]^报道肺鳞癌中的ERCC1表达显著高于肺腺癌。我们以前的研究^[10]^结果显示ERCC1在肺鳞癌及腺癌中的表达无显著差异，本文的研究结果进一步显示ERCC1在NSCLC中的阳性表达明显高于小细胞肺癌患者（*P*=0.039）。Olaussen等^[12]^研究显示，ERCC1表达与肿瘤大小、临床分期无关，与我们的研究结果相一致。ERCC1的表达与肺癌患者预后的关系目前尚未明确，多数研究显示在早期术后患者中ERCC1高表达者生存时间显著延长，可作为指导预后的独立预测指标^[8]^，但也有相反的结果，Azuma等^[13]^研究显示ERCC1阴性的早期肺癌患者比阳性者有更长的无进展生存期。与早期肺癌相反，在接受铂类化疗的晚期NSCLC患者中，ERCC1阳性表达者预示着对铂类化疗耐药，常常导致化疗失败，因此，晚期NSCLC患者ERCC1阳性表达者反较阴性表达者预后差^[12]^。这是因为ERCC1在肿瘤不同的阶段具有不同的作用，通过预防突变，ERCC1可减少癌症的发生或者阻止已经存在的肿瘤进展。因此，对于未进行化疗的早期NSCLC患者，ERCC1是预后良好的标志；而对于晚期NSCLC，若进行化疗则可能导致耐药。因此，ERCC1对于NSCLC患者治疗的选择存在2种情况：早期患者，ERCC1高表达则可以不进行辅助化疗；晚期患者，ERCC1高表达，则不适合应用含铂类为基础的方案化疗，可使用非铂类方案化疗，也可根据*EGFR*基因检测结果选用EGFR通路的TKI抑制剂。我们的研究结果表明ERCC1阳性的肺癌患者预后优于阴性患者，并与生存显著相关（*P*=0.037），这可能是因为我们的病例大多数是接受可能治愈性手术的早期肺癌患者有关。虽然ERCC1表达总体上并非是预后的独立因素，但分层分析发现，ERCC1的表达是影响Ⅰ期和Ⅱ期患者预后的独立危险因素（*P*=0.003），而Ⅲ期患者ERCC1的表达不是影响预后的独立危险因素。另外，Ⅰ期和Ⅱ期ERCC1阳性患者的5年生存率亦显著优于Ⅲ期ERCC1阳性患者。分层分析发现，NSCLC患者中ERCC1阳性患者的5年生存率明显高于ERCC1阴性患者，SCLC患者中ERCC1的表达对生存无显著影响。

GST-pi是人体内一种Ⅱ相代谢酶。其对肿瘤的耐药作用主要由其解毒功能引起，作用机制为：①催化谷胱苷肽（GSH）与亲电子药物如各种烷化剂结合，增加其水溶性，加速其排泄而使药效减低；②清除葸环类药物等产生的自由基，减轻药物自由基对细胞的损伤；③通过直接与药物结合的形式降低药物活性；④GST-pi还具有GSH过氧化物酶活性，可将有毒的过氧化物转变为低毒的醇类物质，即有阻断脂质过氧化物的作用。

NSCLC对化疗的敏感性不如SCLC，其中一个重要的差异就是GST-pi表达的差异。GST-pi在不同肺癌组织类型中的表达目前报道不一。我们以前的研究^[14]^发现GST-pi在肺腺癌中的表达明显高于鳞癌，不同分化程度的NSCLC中GST-pi的表达无明显差异，吸烟者GST-pi的表达低于不吸烟者；Allen等^[15]^的研究也有相似的结论。而王笑新等^[16]^研究发现GST-pi在鳞癌中的表达高于腺癌。我们的研究结果显示GST-pi在肺鳞癌及腺癌中的表达高于SCLC，分层分析显示GST-pi在鳞癌和腺癌中的表达差异无统计学意义（*P*=0.477）；GST-pi在非吸烟患者中的表达明显高于吸烟患者。GST-pi的表达与患者年龄、性别、肿瘤大小、分化程度、淋巴结转移及TNM分期等无关。Hirano等^[17]^的研究显示NSCLC患者接受铂类为基础的化疗疗效与GST-pi的表达有关，GST-pi阴性表达组5年生存率优于阳性组。我们以前的研究^[14]^显示GST-pi的表达与生存无相关，本次研究结果进一步显示无论NSCLC还是SCLC患者GST-pi的表达对生存均无影响（*P*均 > 0.05）。

DNA修复基因的修复能力与肺癌的发病和耐药有关，谷胱甘肽S-转移酶是细胞抗损伤抗癌变的重要解毒系统，我们的研究结果显示ERCC1和GST-pi在NSCLC中表达明显增高并且相关，提示二者在肿瘤形成及铂类耐药过程中可能有一定联系，但是否存在相互调节作用目前尚不清楚，有待进一步研究加以证实。
